# Role of the Intestinal Epithelium and Its Interaction With the Microbiota in Food Allergy

**DOI:** 10.3389/fimmu.2020.604054

**Published:** 2020-12-07

**Authors:** Ayesha Ali, HuiYing Tan, Gerard E. Kaiko

**Affiliations:** ^1^ School of Biomedical Sciences and Pharmacy, University of Newcastle, Newcastle, NSW, Australia; ^2^ Hunter Medical Research Institute, Newcastle, NSW, Australia

**Keywords:** epithelium, microbiota, allergy, anaphyalaxis, intestinal immunity

## Abstract

The intestinal epithelial tract forms a dynamic lining of the digestive system consisting of a range of epithelial cell sub-types with diverse functions fulfilling specific niches. The intestinal epithelium is more than just a physical barrier regulating nutrient uptake, rather it plays a critical role in homeostasis through its intrinsic innate immune function, pivotal regulation of antigen sensitization, and a bi-directional interplay with the microbiota that evolves with age. In this review we will discuss these functions of the epithelium in the context of food allergy.

## Introduction to the Intestinal Epithelial Barrier

The intestinal epithelium lining forms the luminal surface to the external environment of both the small and large intestines. The intestinal epithelium provides a controlled homeostatic system for molecular transit in order to mediate the balance of the multiple functions of the intestinal tract; digestion, immunity and tolerance, and a repository for the various known and unknown symbiotic functions of the microbiota. In order to achieve this fine balance the intestinal epithelium acts as mucosal barrier to micro-organisms while permitting a pathway to protein antigens and small molecule metabolites.

The first layer of physical defense, the mucus layer, plays an important role in reducing adherence of pathogenic microbes while providing a rich-layer of sustenance for slow-growing anaerobic commensal organisms (1, 2). It also acts to shield the host from digestive enzymes and microorganism epithelial penetration. The renewal of the mucus layer (colon consists of an inner more viscous and outer less viscous layer, small intestine a single layer only) is achieved through the secretion of mucus by goblet cells (primarily *via* the *MUC2* gene) (3). The underlying layer of intestinal epithelial cells (IECs) functions as a physical barrier facilitated by tight junctions (4, 5). The intercellular junctions consist of desmosomes, gap junctions, and adherent junctions made up of various integral proteins such as claudins, occludins, and zonula occludens (ZO-1, ZO-2) which operate in concert to maintain the integrity of the epithelial barrier by regulating the paracellular transport of ions, metabolites, and macromolecules (4–7). The tubular crypt and epithelial lining that defines the structure of both the small intestine and colon is continuously regenerated (every 3–5 days) by a population of long-lived Lgr5+ stem cells that reside at the base of the crypt (8). These stem cells give rise to progenitor cells (transit amplifying cells) and multiple differentiated epithelial cell subsets as they migrate through division up the crypt-villus axis before undergoing a program of apoptosis and luminal shedding. Each of these differentiated epithelial cell subtypes possess specific functions in maintenance of the barrier’s digestive, neuroendocrine, and immune functions. These include absorptive cells (colonocytes, enterocytes), goblet cells, enteroendocrine cells, Paneth cells (of the small intestine), tuft cells, and M cells (8). As the epithelium and associated mucus layer provide the first line of defense against microbes they also provide the first line of tolerance against commensal members of the microbiota. In order to achieve this the barrier maintains a hypo-reactivity to microbial ligands in stark contrast to the underlying immune-stromal-rich layer of lamina propria, which is home to the innate and adaptive immune cells with far greater reactivity to commensal and pathogenic microbial ligands (9, 10).

Disruption of the integrity of the intestinal barrier is often associated with inflammatory bowel disease, however, this process is also linked to numerous other local and systemic inflammatory diseases. For example, reduced expression of tight junction proteins leads to a rise in intestinal permeability associated with both local intestinal food allergies as well as systemic allergy in the form of asthma (11–14). In this review, we will discuss the role of the intestinal barrier and IECs in regulating food allergy, as well as specific factors (host and environmental/microbiome) that may create a susceptibility within the epithelial barrier to promote food allergy. Host factors may include changes in protein expression resulting in alterations to the epithelial barrier. This could be either intrinsically programmed in the IECs (genetic or epigenetic) or extrinsically regulated, however, this distinction of etiology remains an open hypothetical question as currently there is little research in this area. We will also discuss how the microbiome closely interacts with the intestinal epithelium and may act as a conduit for changes mediated by an array of exogenous environmental factors including diet.

## IEC Specification and Function in Food Allergy

The intestinal epithelial barrier consists of different IEC subtypes with diverse functional specification interspersed with non-epithelial cell types including intraepithelial lymphocytes. Food allergy is a highly heritable condition (~80%), however, GWAS studies in food allergy have suffered from heterogeneity of disease classification (15). In terms of genetic susceptibility in epithelial genes a recent GWAS demonstrated a strong association between the *SERPINB10* gene and susceptibility to multiple food allergies (16). This gene is known to be expressed by epithelial cells and involved in IL-13-induced transcriptional changes in bronchial epithelial cells, suggesting a possible role for epithelial SERPINB10 in mediating susceptibility to food allergy (17). More work is required to dissect the potential mechanism and interrogate larger GWAS populations pools. Any intrinsic epigenetic changes in the epithelium that promoted or suppressed susceptibility to food allergy would need to be programmed in the long-lived dividing stem/progenitor cells of the crypt base. To date this remains largely unexplored.

Mature enterocytes and colonocytes are polarized epithelial cells lined by tight junctions and responsible for maintaining the integrity of the barrier (4, 5, 18). The apical surfaces of the enterocytes are covered with an array of tiny microvilli increasing the cell surface for absorption and acting as sites of high digestive enzyme concentration (glycosidases, peptidases, and lipases) key to regulating the breakdown and uptake of protein and carbohydrate, food allergens (19). IECs are polarized such that the apical plasma membrane (brush border and microvilli) is distinctly different from the basolateral membrane with different sets of transport proteins at each (19, 20). Overlying the brush border are enzymes that catalyze the final stages in the digestion of proteins, lipids, and carbohydrates. The enzymes at this brush border are tethered to the membrane. One of the key brush border enzymes is enteropeptidase, which catalyzes the activation of trypsinogen (one of the major proteases secreted from the pancreas) into trypsin a key proteolytic enzyme to breakdown proteins into amino acids together with exopeptidases and endopeptidases (19, 20). Amino acids and glucose (from carbohydrate metabolism) are transported across the epithelium against their concentration gradient from the intestinal lumen across IECs by a two-Na^+^/one-glucose (or amino acid) symporter located in the microvilli membranes (19, 20). The Na^+^ ions that are pumped into the cell are pumped out across the basolateral membrane by the Na^+^/K^+^ ATPase with energy provided by ATP hydrolysis. Intracellular glucose and amino acids are pumped across the basolateral membrane into the blood by specific transporters, including GLUT2 (19, 20). In the case of lipid and fat aggregates bile acids facilitate their absorption across the intestinal epithelium. Bile acids contain both hydrophobic and hydrophilic faces. This property allows emulsification of fat into microscopic droplets of micelles, ensuring fat is more easily digested by lipases produced by the pancreas and IECs, while enhancing transport across the epithelium (19, 20). The negative charge imparted on lipids by bile acid adsorption alters electrostatic interaction in the negatively charged mucus, thus facilitating movement of digested lipids through the mucus layer (19, 20).

IECs can express class I and II MHC molecules, thus facilitating a role in non-professional antigen presentation (21), and potentially enabling the direct presentation of protein antigens to lymphocytes that may regulate an allergic response in the intestinal tract. Furthermore, evidence across multiple cell lines demonstrates IEC secreted exosomes contain MHC-II/peptide complexes capable of generating antigen-specific responses in mice (22–24). This suggests a paracrine mechanism by which absorptive IECs may also mediate indirect presentation of food allergens to the immune cells. However, the nature of the induced immune response and the type of T cell polarization generated to IEC-derived MHC-II/peptide complexes is not yet clear as *in vivo* studies have found conflicting results for immunogenic versus tolerogenic T cell responses to ovalbumin in experimental models of food allergy (22, 24). It is likely that the nature of the immune response is dependent on the microenvironment of the lamina propria and inflammatory context of the initial antigen exposure. Whether such a process provides signals for oral tolerance or food allergy in humans remains unclear.

Goblet cells are found scattered in between enterocytes and secrete high-molecular-weight glycoprotein mucins (e.g. MUC2). The dynamic mucus layer formed over the epithelial surface provides a physical and chemical protective barrier for the host against enteric bacteria by acting like a molecular sieve limiting bacterial penetration to the mucosa (25). The small intestine possesses a more porous single layer of mucus compared to the dual colonic layers (1). Mucus often works in concert with secreted IgA through specific binding moieties to immobilize bacteria in the intestinal lumen and allow the removal of microbes through subsequent degradation and renewal of the mucus layers *via* intestinal peristalsis (25, 26). Goblet cells play a critical role in luminal to lamina propria immune cell antigen transfer regulating the balance between tolerance and food allergy and this will be briefly overviewed below (27, 28).

Microfold (M) cells are a rare specialized epithelial subtype that are found overlying intestinal lymphoid tissue, such as Peyer’s patches. These cells play a role in transepithelial antigen transport, delivering foreign antigens and microbes to organized lymphoid tissues within the mucosa of the small intestine and colon (29). Initiation of the mucosal immune response occurs when M cells take up luminal microbes and antigens through the process of phagocytosis, endocytosis, or transcytosis and deliver their payload to dendritic cells located in the lymphoid follicle in the lamina propria (30, 31). Studies have also reported that M cells can express IgA receptor on the apical surface to bind and retro-transport luminal secretory IgA-bound antigens back into Peyer’s Patches, thus creating the capacity for sampling, sensing, and tonal control of foreign antigen load (32, 33).

Paneth cells at the base of intestinal crypt are specialist secretors of antimicrobial peptides (AMPs) including lysozyme, secretory phospholipase A2 (sPLA2), C-type lectin regenerating islet-derived protein IIIγ (RegIIIγ), angiogenin4, cathelicidins, and alpha defensins that help to protect the epithelial barrier and maintain a homeostatic balance with the microbiota (34–38). AMPs are generated when pattern recognition receptors (PRRs), such as Toll-like receptors (TLRs), are stimulated by microbial ligands (such as LPS) and nucleotide-binding oligomerisation domain containing molecules (NODs) are activated by muramyl dipeptides (39). The myeloid differentiation primary response gene MyD88 in Paneth cells plays a critical role in translating this microbial ligand sensing into an AMP response to regulate the balance of abundance of both commensal and pathogenic intestinal microbes (40). Despite this key role in intestinal homeostasis and sensing of foreign antigens a role for Paneth cells in relation to food allergy remains unknown. More studies are needed to address this, in particular as to how indirect regulation of the microbiota by Paneth cells as evolves with age may be a key interface in the relationship between the microbiota and food allergy.

Embedded within the epithelium throughout the gastrointestinal tract are a diverse array of enteroendocrine cells (EECs) (41). EECs, of which there are at least 10 subsets, are essential in regulating physiological processes including appetite, stomach emptying, serotonin controls, and glucose levels by secretion of subset-specific peptide hormones and neuropeptides, such as somatostatin, motilin, cholecystokinin, neurotensin, vasoactive intestinal peptide, and entero-glucagon in response to food ingestion stimuli (42, 43). The arrangement of EECs varies in different parts of the gastrointestinal tract and can be distinguished by “closed cells” and “open cells” (41). EECs in the stomach epithelium are “closed cells” due to lack of direct contact with luminal contents, whereas EECs in the small and large intestine have an “open type” cell arrangement as it has microvilli with direct luminal contact. The secretion of hormones by EECs in the small intestine are tightly regulated by the dietary nutrients absorbed, while EECs in the colon may have a greater propensity to respond to a range of microbial products (41). The abundance of EECs in food allergy has to our knowledge not yet been characterized. There are also no studies on a functional link between EECs and IgE-mediated food allergy, however, lower numbers of EECs have been associated with irritable bowel syndrome (IBS) patients with food hyper-sensitivities, and dietary intervention studies to remove food triggers and reduce symptoms induce a normalization in Chromogranin A-positive EECs in colonic biopsies of IBS compared to healthy controls (44–46). Given the increasing recognition that a significant proportion of IBS may represent a type of non-IgE localized atypical food allergy there may be a key role for factors released by EECs in this type of non-classical food allergy. In a seminal prospective study of 108 IBS patients, 70% of those endoscopically challenged with four specific food components demonstrated immediate disruption of the intestinal barrier characterized by fluid extravasation, changes in permeability and tight junctions, as well as increased eosinophil degranulation (47). Although an emerging area with more evidence and interventional studies required, a role for EECs in localized atypical non-IgE food allergies is an important avenue for future research.

Tuft cells are a rare chemosensory epithelial subtype known for their importance in expulsion of parasitic helminths and generation of a strong Type 2 immune response as the exclusive source of IL-25 in the intestine (48–50). These cells promote the outgrowth of Type 2 innate lymphoid cells (ILC2s) and drive eosinophilia in the intestinal tract, and are induced to proliferate from stem/progenitor cells in the crypt through an IL-13-dependent feed-forward mechanism (48–50). Despite Tuft cells being a clear mediator of Type 2 immunity in the gut there has been minimal evidence so far for their role in food allergy. A recent study from *Leyva-Castillo et al.* was able to demonstrate that mechanical skin injury-induced IL-33 release triggered a concomitant increase in small intestinal mast cells and this drove enhanced anaphylaxis following oral challenge (51). This work convincingly showed that the increase in small intestinal mast cell numbers was elicited by an expansion of IL-25-producing intestinal Tuft cells driving the proliferation of IL-4/IL-13-secreting ILC2s in the intestine. The direct effect of Tuft cells (through genetic deletion) on food-induced anaphylaxis was not assessed. However, this study represents the first evidence for a role of Tuft cells in food allergy.

Detailed studies focusing on the contribution of individual IEC subtypes and their secreted products to the pathogenesis of food allergy are likely to emerge over coming years due to the advent of new technologies. The use of intestinal organoids from primary human tissue along with single cell sequencing tools will enable a greater resolution for the functional dissection of IEC subsets (52). Such findings can then be translated into gene knockout studies in mouse models of food allergy, and even orthotopic transplantation of organoids into immune-deficient mice. Most *in vitro* studies to date have relied on colorectal cancer cell lines in two-dimensional monolayers and although this has provided important and valuable insights many of the characteristics of immortalized monolayers are distinctly different from primary human epithelial cells generated from intestinal stem cells, including their metabolism and ability to generate differentiated cell subsets (53–56).

## Role of the Intestinal Epithelial Barrier in Regulating Protein Antigen Passage, and Allergic Responses to Food Antigens

**Figure 1:**
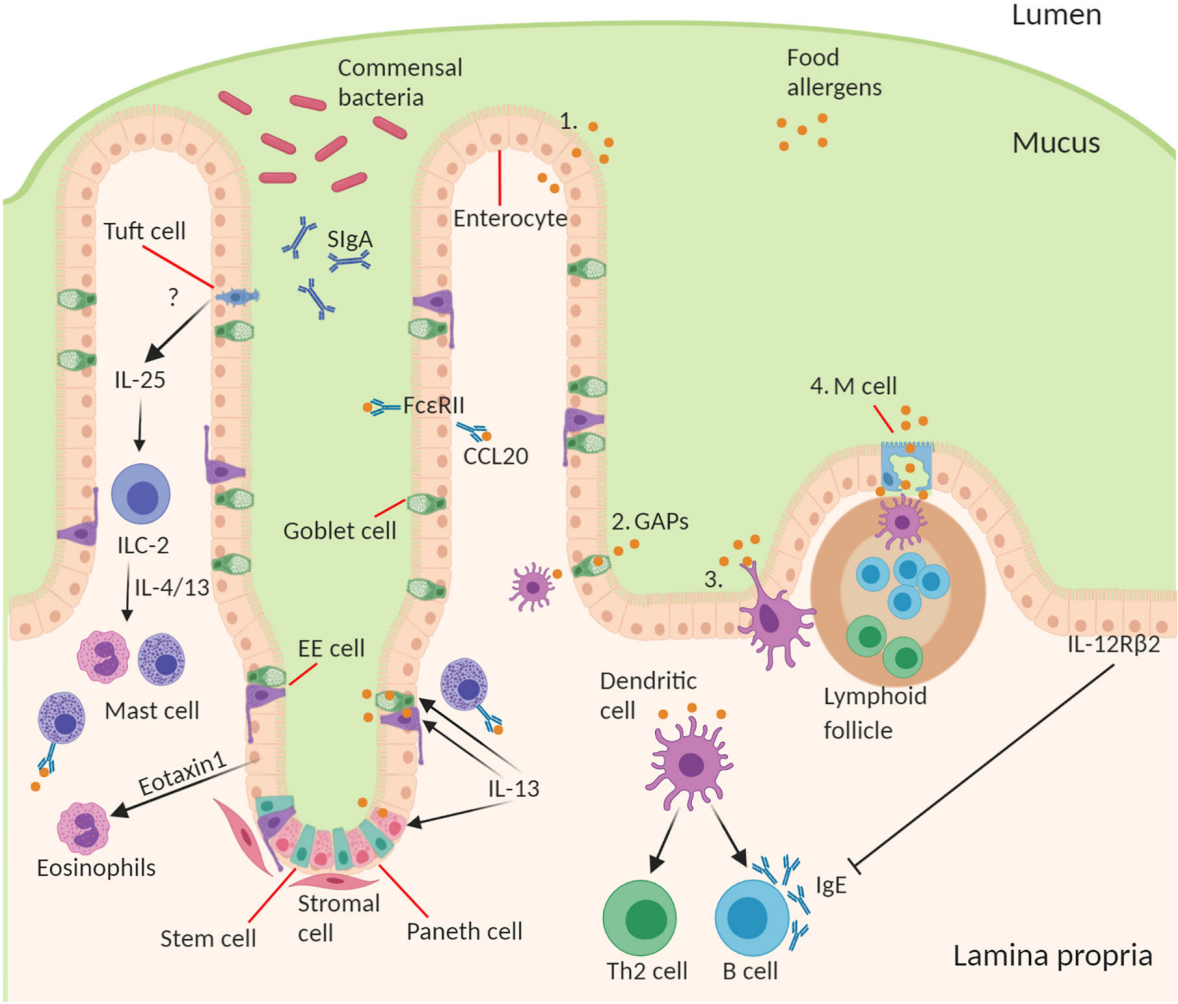
Structure and cell type specification of intestinal epithelium and function in antigen uptake. The intestinal epithelial barrier consists of stem cells, Paneth cells (small intestine), goblet cells, tuft cells, enteroendocrine (EE) cells, enterocytes (small intestine)/colonocytes (colon), and microfold (M) cells overlying lymphoid follicles. *Route of antigen uptake:* (1) paracellular transport of food allergen, (2) goblet associated antigen passages, (3) direct antigen uptake by dendritic cells (DCs) stretching a dendrite into the lumen between the epithelial intercellular space, (4) M cell mediated antigen transcytosis. DC present food antigen to lymphocytes to T cell differentiation into Th2 cells and B cell isotype class switching to produce allergen-specific IgE. *Contribution of IECs to amplifying food allergy*: IgE complexed with food antigen can transcytose across intestinal epithelial cells *via* FceRII, this process stimulates CCL20 release. Tuft cell produce IL-25 driving the expansion of ILC2s and their production of IL-4 and IL-13, which in turn drive mast cell and eosinophil activation. Epithelial cells produce Eotaxin-1, a chemoattractant for eosinophils. During an allergic response IL-13 acts on secretory cells like Paneth cells, EE cells, and goblet cells to increase antigen uptake and presentation to IgE-bearing mast cells. Mechanism of inhibition of allergic response by IECs through activation of basolateral expressed IL-12Rβ2.

It is important for the intestinal epithelial barrier to control different types of luminal antigens being translocated in order to maintain homeostatic control and to guide mucosal immune responses in the fine balance between tolerance and sensitization (57). There are several specialized pathways involved in the transport of antigen from the lumen across epithelium to underlying lamina propria immune cells (28, 58–60). The route through which antigens cross the epithelial barrier may have a profound influence on the type and scale of immune response generated (**Figure 1**).

IECs exist in close contact with T cells and dendritic cells in the underlying lamina propria to facilitate an antigen flow through the professional antigen presenting cells (APCs), and as outlined the IECs can themselves function as non-professional APCs. The epithelial layer plays an important role in protecting the mucosal immune system from excessive exposure to immunogenic proteins. This protection of the epithelium is maintained by the formation of tight junctions (TJs) between cells (61, 62). TJs are a multi-protein complex that forms a selectively permeable seal between adjacent epithelial cells near the apical surface (61, 62). Metabolites, ions, and water can pass through the intercellular space between cells using a paracellular pathway, which is regulated by TJ proteins such as claudins, occludins, and ZO-1. However, any alteration in this passive paracellular transport in the form of leaks can allow macromolecules like food antigens to cross the epithelial barrier increasing the exposure to antigen that could lead to allergic sensitization. Several *in-vitro* studies using IECs have shown that specific dietary components can alter TJ proteins and overall barrier integrity (61, 62). Gliadin, a wheat protein, can interfere with the interaction between occludins and ZO-1, leading to increased monolayer permeability (63). Other dietary components, such as capsinoids in chili, have also demonstrated capacity, *in vitro* at least, to alter paracellular flux and decrease TJ barrier integrity (64, 65). This process of IEC paracellular transport is dysregulated *in vivo* in the small intestine during oral antigen challenge in pre-sensitized mice, leading to the development of secretory diarrhea (66). The leaky jejunoileal epithelium is dependent on a rapid intestinal epithelial Cftr-dependent Chloride ion secretory response associated with increased paracellular permeability from loss of intercellular claudin-1, 2, 3 and 5, E-cadherin, and desmosomal cadherins. Similarly, the loss of water channel expression (Aquaporin 4 and 8) in IECs during an oral challenge in a pre-sensitized setting also likely contributes to secretory diarrhea (67). In this way the intestinal epithelium also creates a feed-forward loop where a leaky barrier to antigen likely amplifies the allergic response.

IECs constitutively express the CD23 (Fc epsilon RII) receptor for IgE on their membranes, which enables transcytosis of IgE and capture of IgE/antigen complexes to transport it across the epithelium from the lumen to lamina propria (68, 69). This IgE/antigen transport process is increased in a pre-sensitized setting of food allergic patients and can trigger IECs to secrete CCL20, thus acting to amplify the allergic response to food antigens (68–70). The intestinal epithelium can also release cytokines and chemokines to both amplify or suppress the allergic response. For example, IECs are a major source of eotaxin-1 that drives eosinophil recruitment to the gut in food allergy (71). Epithelial Type 2-inducing cytokines, such as IL-25, IL-33, and TSLP, are important drivers of inflammation in food allergy, however, most experimental studies have relied on whole body knockouts or systemic antibody-mediated neutralization in mice (72–75). While IECs can certainly produce IL-25 and TSLP, the importance of IECs as the cellular source of these cytokines in food allergy remains unclear because IEC-specific conditional-deletion studies remain limited, and some food allergy models rely on skin keratinocytes to produce these cytokines through epicutaneous sensitization (73, 74). IECs also express the IL-12Rβ2 chain on their basolateral surface in proximity to underlying immune cells, and activation of this receptor by IL-12 (a Th1 immune response-inducing cytokine) leads to a reduction in serum IgE and food anaphylactic responses (76).

M cells on the apical surface of intestinal lymphoid follicles mediate antigen uptake and transcytosis across the intestinal epithelium (58). These cells are specialized for uptake of particulate antigens and intact microorganisms from the lumen facilitating transcellular transport to the underlying dendritic cell rich sub-epithelial dome where they are can initiate or amplify an immune response (77). In addition to non-specific uptake of antigens there is also a receptor-mediated transport that occurs through M cells. Various enteric pathogens such as *Salmonella enterica* can enter into the host through M cells by the means of GP2-FimH interactions (78). IECs transport IgA into the intestinal lumen where these antibodies contribute to barrier function by excluding uptake of antigens as well as microbes. M cells are important in the production of these IgA antibodies given their antigen sampling, intimate proximity to lymphoid follicle B cells, and co-dependency of M cells and IgA induction on RANKL (79–81). Thus, M cell-mediated antigen uptake may help to maintain control over gut immune homeostasis and microbial diversity through an IgA-dependent feedback loop. Moreover, this function of M cells may play an important role in the genesis of food allergy. Using a peanut mouse model of food allergy *Chambers et al.*, showed that in the pre-sensitized gut epithelium ingested peanut proteins were present inside the M cell cytoplasm with negligible presence in absorptive enterocytes (82). These antigen sampling M cells allowed passage of the protein to the mucosal immune cells of the sub-epithelial dome.

Intestinal goblet cells also perform a critical role in the uptake of luminal antigens and provision to the immune cells (28). Goblet cells are present throughout the intestinal crypts and villi (aside from the follicular associated epithelium) and their capacity to sample the lumen provides a key sentinel function. In seminal work McDole et al., used *in-vivo* two-photon imaging experiments to demonstrate that goblet cell-associated antigen passages (GAPs) were able to deliver a diverse set of antigens from the intestinal lumen to CD103^+^ lamina propria dendritic cells (DCs) (28). The role of GAPs in transferring protein antigens to tolerogenic DCs, that migrate to mesenteric lymph nodes and promote the development of T-regulatory (T regs) cells, may help facilitate oral tolerance. Aside from this active control by GAPs over antigen passage to DCs, the epithelium can also release TGF-β, and metabolize vitamin A into retinoic acid to induce the development of CD103^+^ tolerogenic DCs (83–85). A high frequency of GAPs are observed in the small intestine in mice and the formation of these passages has also been noted in human small intestinal tissue with responsiveness to acetylcholine. In contrast, at steady state in the colon goblet cells have a dramatically diminished capacity to form GAPs due to inhibitory stimuli from a high microbial load (27). However, antibiotic-mediated acute disruption of the microbiota facilitates the formation of colonic GAPs enabling delivery of luminal antigens to colonic DCs (86). In the context of established food allergy IL-13 can act to dramatically increase the number of these small intestinal GAPs, and expand the type of secretory cells that form these passages to include Paneth cells and EECs (87). This process drives anaphylaxis through antigen transfer to mucosal mast cells.

The fourth mechanism by which luminal antigen can be sampled involves direct action of DCs. Aside from uptake of antigen indirectly provided by epithelial cells, DCs can also directly sample the gut lumen through extending trans-epithelial dendrites (TEDs) (60, 88). DCs on the basolateral side of the epithelium can extend these TEDs across the TJ complexes to sample antigen and microbes (60, 88). Moreover, DCs can express their own TJ proteins, such as occludin, claudin, and ZO-1, to interact with epithelial cells to permit sampling of luminal antigens without disruption of the epithelial layers (60, 88). The ability of DCs to sample the small intestinal lumen for antigen through TEDs is driven by CX_3_CR1/fractalkine, a transmembrane chemokine expressed at the surface of IECs (59). CX3/CR1-deficient mice show impaired ability of DCs to sample bacteria from the lumen. CX3/CR1^+^ DC TEDs act to drive inflammatory responses to pathogens but it remains less clear whether this process is involved in food antigen sensitization or oral tolerance (89, 90).

The sampling and sensitization of food allergens occurs predominately in the small intestine and similarly the localized inflammatory responses, intestinal permeability, and feedback amplification of this process are manifested primarily at the jejunal-ileal region. However, it remains entirely possible given the migratory nature of immune cells that tolerogenic or pro-inflammatory changes, such as those orchestrated by the microbiota, in the colon may impact the small intestinal tract and vice versa.

## Interplay Between IECs and the Microbiome in Food Allergy

**Figure 2:**
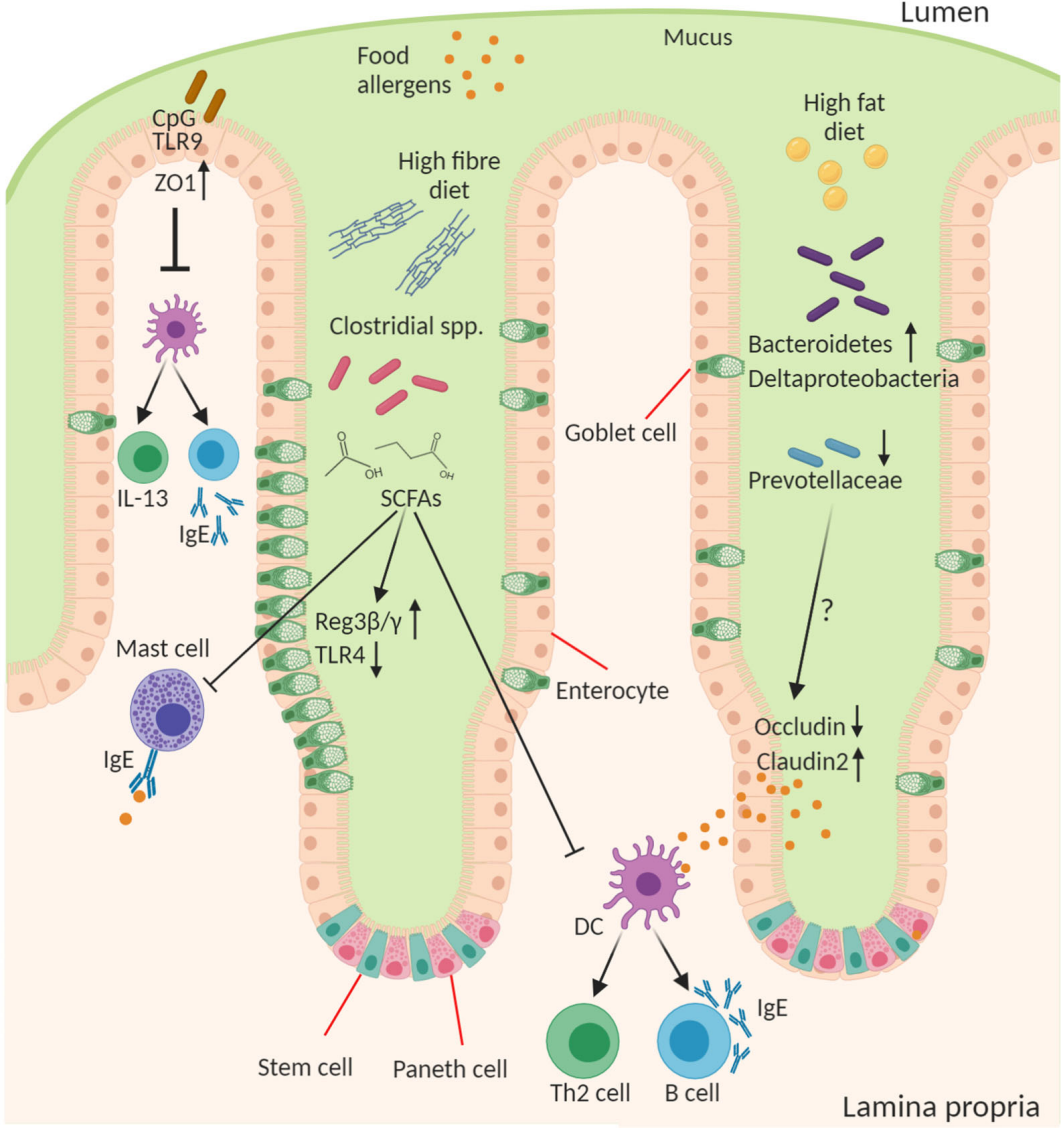
Role of the microbiota in epithelial regulation of food allergy. Diet and the intestinal microbiota interact to produce the outgrowth or loss of certain bacterial genera in food allergy this can result in altered concentration of microbial ligands, such as TLR ligands, and small molecule metabolites that stimulate host epithelial and immune responses. CpG oligonucleotides from bacterial DNA bind epithelial TLR9 to increase expression of ZO-1 improving epithelial tight junction barrier integrity to antigen and reducing cytokine production from immune cells. A high-fiber diet leads to outgrowth of many fermenting Clostridial species and enable their production of short chain fatty acids (SCFAs), in particular butyrate, that act on the epithelium to induce goblet cell hyperplasia, increase antimicrobial proteins (Reg3β and γ), and reduce TLR4 expression. SCFAs also inhibit mast cell activation and favor the development of tolerogenic dendritic cells (DCs) and regulatory T cells and suppress the inflammatory DC-Th2-IgE pathway. A high-fat diet leads to increase in Bacteroidetes and Deltaproteobacteria and reduction in abundance of Prevoltellaceae. This microbial dysbiosis drives increased barrier permeability and antigen transfer to lamina propria cells by increasing Claudin2 and decreasing Occludin tight junction expression. The soluble microbial factors responsible for this epithelial change remain to be discovered.

The importance of the microbes in food allergy is a concept that first arose in the context of the “hygiene hypothesis” which posited that a loss of microbial exposure was responsible for a skewing of the immune response and increased susceptibility to allergies in “westernized” countries (91). This exposure may be altered by lifestyle changes including antibiotics, public hygiene, dietary changes, migration from rural/farming to urban communities, and higher rates of Caesarean birth and formula feeding. Since the advent of sequencing technologies to study the microbiome and gnotobiotic tools for its manipulation in experimental systems this area has received renewed attention with a reductionist approach to explore this relationship. Researchers have begun to examine how microbes and their molecules interact with the host, which has led to the development of a new iteration of the “hygiene hypothesis,” which suggests that a balanced intestinal microbiota characterized by diverse Firmicutes is necessary for intestinal health and loss of this balance increases susceptibility to food allergy (**Figure 2**). Although there is a dominant heritable component to food allergy, there is obviously a significant role for environmental factors, including “westernized” lifestyle changes, that predispose to disease. All these lifestyle factors from antibiotics, to diet, to urbanized living converge to have an impact on the composition and function of the intestinal microbiota. Moreover, the intestinal microbiota is itself highly heritable and so likely plays a role in the substantial heritability of food allergy. Indeed the microbiome may help to explain the significant “heritability gap” so commonly observed for many diseases, including food allergy, where the predicted heritability derived from twin and sibling studies is not matched by that predicted by GWAS of host genetics. The microbiome heritability in early life is derived from the impact of familial genetics on microbiome composition, sharing a common environment especially during development and childhood, and the strong maternal influence on the microbiota from birth to breastfeeding (92). The large TwinsUK population study revealed the strong influence of shared host genetics on microbiota taxa including with specific microbial genera that play a role in basic human traits, such as BMI (93). Furthermore, host gene variants such as those in *APOA5*, *LCT*, *NOD2*, and *FUT2* have also been shown to profoundly impact the composition of the microbiome (92, 94). But perhaps the most important influence on the early childhood microbiota, and its strongest source of heritability, are maternal factors in vertical transmission including diet, delivery mode, and breastfeeding (95–97). In the following section we will discuss how the microbiota contributes to the predisposition to food allergy.

One of the most widely studied mechanisms for microbiota to host signaling is *via* the TLR ligand-PRR recognition system. Of the TLR ligands analyzed the strongest data functionally linking to allergy appears to be attributed to TLR4 and TLR9. Studies in T84 colorectal cell line monolayer cultures have shown that stimulation of TLR9 reduces inflammatory cytokine production in co-cultures with PBMCs from food allergy patients (98). The location of TLR9 activation at the apical surface, rather than basolateral, may be key to driving this more tolerogenic response (99). Unmethylated versus methylated CpG/TLR9 signaling as the dominant signal to IECs may play a role in distinguishing a healthy microbiota *versus* an invading bacteria and driving the generation of tolerogenic DCs and Tregs (100, 101). Furthermore, TLR2 and TLR9 on IECs have been shown to protect against barrier disruption by enhancing ZO-1 expression (102, 103). In contrast, activation of TLR4 had an opposing effect. Interestingly, wheat α-amylase trypsin inhibitors, a recognized plant-derived food allergen, activates TLR4 (104).

A large Twins cohort study (>2,700 individuals) recently revealed that the top three diseases most strongly associated with changes in the microbiota are inflammatory bowel disease, Type 2 diabetes, and food allergy (105). The most prominent association in this disease analysis indicated a negative association of food allergy with the abundance of Prevotellaceae. In mice a reduction in *Firmicutes* spp. and increase in *Proteobacteria* spp. (an observation common to many chronic inflammatory diseases) has been associated with an aggravated response to food antigen, and colonization of germ-free (GF) mice with the fecal microbiota of healthy infants, rich in *Bifidobacterium* spp. and *Bacteroides* spp., protected against the development of cow’s milk allergy (CMA) (106, 107). There is evidence that human milk oligosaccharides, which increase the abundance of *Lactobacillus* and *Bifidobacterium* strains may act to suppress the pro-allergic cytokine release by IECs, suggesting a possible mechanistic role of IECs underlying the negative correlation between breast feeding and development of food allergy (108, 109).

Work from *Stefka et al.*, demonstrated that treatment of mice with antibiotics led to enhanced IgE responses to peanut allergen with similar observations in GF mice (110). The authors showed the key bacterial genera mediating the microbiota-driven allergy suppression were the Clostridium clusters XIVa, XIVb, and IV. These clusters are primarily fiber-fermenting bacteria that produce short chain fatty acids (SCFAs), such as butyrate, propionate, and acetate. Mono-colonization of GF mice with these bacteria increased Foxp3+ Tregs, IgA, and IL-22 and reduced mast cell activation. In the epithelium these bacteria enhanced levels of AMPs (Reg3β and γ) and increased numbers of goblet cells. In follow-up work the authors extended these observations to show that GF mice colonized with fecal bacteria from healthy, but not CMA, infants were protected against anaphylactic responses to a cow’s milk allergen (111). By correlating taxonomic and host ileal signatures from colonized GF mice, the Clostridial species *Anaerostipes caccae* was identified as a key candidate. Gnotobiotic experiments revealed that the butyrate-producer *A. caccae*, which was diminished in CMA, was able to protect against the allergic response to food. It was previously demonstrated that dietary supplementation with fiber or butyrate was able to induce similar oral tolerance (112). Butyrate is of particular interest among the fiber fermentation SCFA products because it is known to act directly to suppress many inflammatory responses including mast cell activation, induce AMPs, IL-22, and goblet cell hyperplasia, as well as induce Treg proliferation through histone deacetylase inhibition and GPR43/GPR109A agonism (112–116). Butyrate is also an important metabolic energy source for differentiated colonocytes (56). Further support of these findings for a protective effect of butyrate-producing species is provided by the observation that *Clostridium butyricum*, which produces high levels of butyrate from granulose starch, can suppress TLR4 expression by colonic IECs, potentially improving barrier integrity (117). Despite these discoveries we are likely only now just scratching the surface of this complex interaction with the host, and getting to a point where it’s clear there are many more unknowns than knowns in regards to microbiota-derived molecular signals.

If a “healthy” high-fiber diet can reduce susceptibility, then recent evidence suggests the converse might also be true with a “westernized” diet. *Hussain et al.* recently demonstrated that a high fat diet (independent of obesity) induces changes in the mouse intestinal microbiota that lead to enhanced susceptibility to food allergy (118). Not surprisingly the diet led to profound changes in bacterial communities with increases in *Desulfovibrionaceae* (Deltaproteobacteria), and *Rikenellaceae* (Bacteroidetes) and a reduction in the abundance of the *Muribaculaceae* and *Prevotellaceae*. Interestingly, the latter correlates with the microbiome results of the food allergy analysis in the twins cohort study (105) and likewise in a separate birth cohort study with maternal carriage of *Prevotella* being protective against infant risk of food allergy (119). This microbial dysbiosis altered the intestinal epithelial barrier to facilitate increased passage of protein allergen correlating with reduced expression of occludin and increases in the channel forming TJ protein claudin-2 (118). However, the mechanism and molecular factors responsible for this microbiota-induced change in IECs remains unclear.

The microbiota has the potential to produce an enormous array of products with bioactivity on the human epithelium and immune system, most of which remains to be discovered. Many such compounds may impact susceptibility to food allergy. For example, the microbiota can metabolize tryptophan into ligands for aryl hydrocarbon receptors expressed on epithelial and immune cells, transform primary into secondary bile acids with effects on IECs and Tregs, produce monohydroxy fatty acid 12,13-diHOME linked to asthma, and the intestinal microbiota can even produce histamine in asthma patients (120–123). The reasons why some bacterial species are more closely correlated with food allergy than others are of interest for the development of prevention strategies, especially probiotics. However, on the flip-side some bacteria are also capable of producing proteins that themselves act as allergens. In patients suffering from allergic disorders such as asthma, atopic dermatitis, or nasal polyposis, *Staphylococcus aureus* colonization appears more frequently (87, 90, 87%, respectively), in contrast to (20–50%) colonization of healthy adults (124–126). Furthermore, cell culture supernatants of *S. aureus* induce mast cell degranulation *via* δ-toxin (127). Many asthma, nasal polyposis, rhinitis, and atopic dermatitis patients demonstrate Staphylococcal enterotoxin and fibronectin binding protein-specific IgE in their serum, and so the ability of this group of bacterial proteins to act as allergens has been well characterized (127–129). Interestingly, skin colonization with *S. aureus* is also correlated with the onset of food allergy (peanut and egg) in infants, independently of the severity of atopic dermatitis (130, 131). Furthermore, as breast feeding is negatively associated with risk of food allergy it is of interest that cessation of this feeding has recently been found to diminish the abundance of *Staphylococcus* species in the infant fecal microbiome (132). Despite these findings there is currently very little evidence for a direct role of specific bacterial proteins, including Staphylococcal enterotoxins, in IgE-mediated food allergy. This is primarily due to a lack of studies to directly address this possibility. Given the strong associations of food allergy with changes in the intestinal microbiome this is an area that requires further investigation. Such future studies would necessitate a combination of *in silico* prediction tools to explore the microbiome based on algorithms using the primary and secondary structure of known allergens. This would need to be followed by empirical validation through sera bacterial protein-specific IgE assessment using ELISA, immunoblot, or peptide arrays.

Future identification followed by functional analysis of bacterial products and specific probiotic organisms will be critical to furthering our understanding of the role and therapeutic potential of the microbiota in regulating the intestinal epithelium in food allergy.

## Potential Treatments Targeting the Intestinal Epithelium

Any new therapies for food allergy are likely to be combinatorial with oral immunotherapy (OIT) either to a specific food, such as peanuts, or to multiple-food OIT protocols. Monoclonal antibodies targeting IL-25 and TSLP are in various stages of clinical development. Tezepelumab (anti-TSLP) has shown promise in treating asthma and is now being trialed in other allergic diseases, including atopic dermatitis (133) (NCT03809663). Changes in IL-25-producing Tuft cells in patients with food allergy remain unclear and require further clinical analysis before a clinical target would be valid. Given the efficacy of anti-TSLP in other allergic diseases, the link to food allergy through the skin and atopic march, and promising evidence from experimental mouse models, this therapeutic option would seem to be a future target worth considering. By combining this with OIT it may maximize efficacy while reducing treatment length of costly biologics. Targeting TLR9 using synthetic analogues with OIT may provide another avenue to harness the epithelium for the treatment of food allergy. TLR9 agonism showed promise in reducing allergic asthma symptoms in Phase 2 trials, however, this approach failed to produce efficacy when used in the more uncontrolled population of moderate-to-severe asthmatics (134, 135). Acute targeting of GAPs during early stages of life through localized delivery of a muscarinic acetylcholine receptor 4 agonist may provide another avenue for induction of oral tolerance or treatment of children at risk of heritable food allergies (90). Evidence from mouse models also suggests that proper synchronization of maternal breastmilk with infant development may also act to prevent an imbalanced immune response to dietary antigens (136). Potentially this an important consideration for infants unable to receive maternal breastmilk and provided instead with a donor source. The use of a prebiotic starch diet, or defined anaerobic Clostridial probiotic consortia, or metabolites such as butyrate may better harness the microbiota to promote oral tolerance to allergen. Perhaps a combinatorial treatment consisting of a high-fiber substrate with a butyrate-producing taxa, and butyrate metabolite may act to feed-forward amplify this effect. Such a treatment would likely need to be applied within the first 3 years of life before the microbiota reaches maturation.

## Conclusion

The intestinal epithelial barrier plays a critical role in maintaining a state of guarded tolerance to the luminal milieu of dietary and microbial antigens. IECs are key to regulating food allergen uptake and presentation to the immune system. The bi-directional interplay between IECs and the microbiota likely plays a critical role in setting the tonal control over allergic sensitization *versus* tolerance. Greater molecular mechanistic understanding of this process, in particular on the microbiome, will be key to finding potential new combinatorial therapies for food allergy.

## Author Contributions

HT and AA contributed equally to the research and writing of the manuscript. GK conceived, co-wrote, and edited manuscript. All authors contributed to the article and approved the submitted version.

## Funding

Funding provided by NHMRC Australia APP1162666 and APP1146524.

## Conflict of Interest

The authors declare that the research was conducted in the absence of any commercial or financial relationships that could be construed as a potential conflict of interest.
